# Prognosis prediction of procalcitonin within 24 h for acute diquat poisoning

**DOI:** 10.1186/s12873-024-00975-2

**Published:** 2024-04-14

**Authors:** Cheng He, Liguo Liang, Yu Zhang, Tianyi Wang, Rongyang Wang

**Affiliations:** Emergency Department of Nanyang Traditional Chinese Medicine Hospital, 473003 Nanyang, Henan China

**Keywords:** Procalcitonin, Acute diquat poisoning, Prognosis, Retrospective study

## Abstract

**Background:**

To explore the predictive value of procalcitonin (PCT) within 24 h after poisoning for prognosis of acute diquat poisoning.

**Methods:**

This retrospective study included acute diquat poisoning patients in the Nanyang City Hospital between May 2017 and July 2021.

**Results:**

Among the 45 patients included, 27 survived. The maximum PCT value within 24 h after poisoning was significantly higher in the non-survival patients [9.65 (2.63, 22.77) vs. 0.15 (0.10, 0.50) µg/mL, *P* < 0.001] compared to the survival patients. The area under the ROC curve (AUC) indicated that the maximum PCT value within 24 h had a good predictive value (AUC = 0.905, 95% CI: 0.808-1.000) compared to ingested quantity (AUC = 0.879, 95% CI: 0.776–0.981), serum creatinine (AUC = 0.776, 95% CI: 0.640–0.912), or APACHE II score (AUC = 0.778, 95% CI: 0.631–0.925). The predictive value of maximum PCT value within 24 h was comparable with blood lactate (AUC = 0.904, 95%CI: 0.807-1.000).

**Conclusions:**

The maximum PCT value within 24 h after poisoning might be a good predictor for the prognosis of patients with acute diquat poisoning.

## Background

Diquat, also known as 1,1’-ethylene-2,2’-bipyridylium cation dibromide, is a herbicide used to control weeds and aquatic plants. It has a molar mass of 344.05 g/mol and is commonly found in a 20–25% aqueous solution. It easily absorbs in the body and is distributed to various organs but does not accumulate in lung tissues [1]. Inhalation of diquat can lead to respiratory distress, inflammation, and pulmonary edema [2], with common symptoms include nausea, vomiting, abdominal pain, and diarrhea [3]. Unfortunately, there is no specific antidote for diquat poisoning. Due to stringent pesticide regulations in many developed countries, the prevalence of diquat poisoning is relatively low; however, due to limited education, training, and safety regulations, its prevalence may be high in some developing countries [4]. Cases where more than 50 mL of diquat was ingested have much higher mortality rate, resulting in an overall fatality rate of 60% [5]. Predicting the prognosis in diquat poisoning is important for clinical decision-making, patient counseling, resource allocation, and assessing its potential long-term impact on patient’s quality of life [6].

Procalcitonin (PCT) is primarily synthesized and secreted by thyroid C cells. It is an inactive precursor of calcitonin, a glycoprotein composed of 116 amino acids, which includes calcitonin, calcitonin gene-related peptide, and N-terminal fragments. PCT has been widely utilized as a novel inflammatory marker to predict prognosis in various clinical conditions, including sepsis [7, 8], pneumonia [9], bacterial infection [10], and acute respiratory distress syndrome [11]. Its concentration in blood is generally elevated under certain conditions, such as bacterial infection and pesticide poisoning, such as paraquat [12]. However, its use in predicting prognosis in diquat poisoning is not well-established.

In China, previous studies have been conducted to evaluate the prognosis of diquat poisoning patients [3, 13-16], examining factors such as the time between poisoning and admission, diquat concentration in the blood and urine, creatinine levels, poisoning index, and acute physiology and chronic health evaluation II (APACHE II) score [17-19]. According to the *“Expert Consensus on Diagnosis and Treatment of Acute Diquat Poisoning” *[20], it is recommended to consider detailed patient history, toxicological analysis, and combined detection methods for prognosis assessment. Indicators suggesting a poor prognosis include the consumption of more than 12 g diquat cations (equivalent to more than 112.20 mL of a 20% commercial product), severe systemic inflammatory response within 24 h, and the occurrence of multiple organ failure. To the best of authors’ knowledge, no study has used PCT in predicting prognosis in diquat poisoning.

Therefore, this study aimed to explore the predictive value of PCT within 24 h for prognosis of acute diquat poisoning.

## Methods

### Study design and patients

The retrospective study included acute diquat poisoning patients who were treated in the Emergency Department and Poison Center of Nanyang City Hospital, Nanyang, China, between May 2017 and July 2021. The inclusion criteria of patients were: (1) Orally ingested diquat; (2) Hospitalized within 24 h of poisoning. The exclusion criteria of patients were: (1) History of infection, trauma, sepsis, or other relevant diseases; (2) Incomplete clinical data records. The diagnosis of acute diquat poisoning was confirmed by testing urine collected at the time of patient consultation using the bicarbonate and dithionite test, as well as considering the ingestion history of patient. All patients received gastric lavage, cathartic treatment, blood perfusion, continuous renal replacement therapy (CRRT), corticosteroids, and symptomatic treatment. The Nanyang Traditional Chinese Medicine Hospital Ethics Committee approved the study (No. 202,219), and the requirement of informed consent from patients was waived for the retrospective nature.

### Data collection

Demographic characteristics (age and gender), as well as clinical characteristics including the amount ingested, time from ingestion to admission in the hospital, serum creatinine, blood lactate, APACHE II score and maximum serum PCT concentration within 24 h after admission were collected. All these information were obtained from the clinical medical record system of the hospital. The patients’ prognosis were evaluated 30 days after admission. Patients who experienced worsening conditions, treatment abandonment, or death within 30 days were classified as non-survival group (poor prognosis). Conversely, those who achieved recovery or maintained stable vital signs within the same period were classified as survival group (good prognosis). The worsening condition was defined as a further deterioration in respiratory, circulatory, or renal function, accompanied by extremely unstable vital signs.

Most of the patients we treated came from districts and counties with large oral doses, and only a few cases with minor inhalation and skin exposure during spraying pesticides were found to be negative for difenoconazole except for local skin lesions, no systemic damage, therefore not included in the statistics.

Among the 10 patients who were poisoned with 100 milliliters of 20% Diclofenac solution, PCT showed an increasing trend. Among them, 6 patients had PCT above 1.12 ng/ml, and 3 of the 4 patients with PCT above 5.9 ng/ml died. Due to the small sample size of the cases, they were not separately counted and compared.

### Statistical analysis

The statistical analysis was conducted using SPSS 19.0 software (IBM Corporation, Armonk, New York, USA). The normality of continuous variables was examined by Shapiro-Wilk test. The continuous variables that confirmed to normal distribution were expressed as mean ± standard deviation (SD), and compared by independent sample *t*-test. The continuous variables with skewed distribution were expressed as median (P_25_, P_75_), and compared by the Mann-Whitney U test. The categorical variables were expressed as n (%) and compared by the chi-square test. Receiver operating characteristic (ROC) curves were constructed to analyze the area under the curve (AUC), sensitivity, and specificity for the predictive value. A two-sided *P* < 0.05 was considered as statistical significance.

## Results

A total of 45 patients were included in the study, including 18 in the non-survival group and 27 in the survival group. The results showed significant differences in age, ingested quantity, and maximum PCT value within 24 h after poisoning between the non-survival and survival groups. Specifically, all cases in the non-survival group had ingested > 100 mL of diquat, the majority of cases in the survival group had quantities below 100 mL. The maximum PCT value within 24 h after diquat poisoning was significantly higher in the non-survival patients [9.65 (2.63, 22.77) vs. 0.15 (0.10, 0.50) µg/mL, *P* < 0.001] compared to the survival patients. However, no significant differences were observed in gender and time from ingestion to admission between the non-survival and survival groups **(**Table 1**)**.

**Table 1:** Demographic information and clinical characteristics of patients

Characteristics	Non-survival group (*n* = 18)	Survival group (*n* = 27)	*P*
Gender, N (%)			0.329
Male	10 (55.56%)	11 (40.74%)	
Female	8 (44.44%)	16 (59.26%)	
Age (Years), mean ± SD	51.28 ± 20.08	37.44 ± 17.05	0.017
Ingested quantity (mL), M (P_25_, P_75_)	200 (100, 200)	50 (30, 100)	< 0.001
Time from ingestion to admission (hours), M (P_25_, P_75_)	5 (3, 8.75)	4.00 (3, 7)	0.935
Maximum PCT value within 24 h after poisoning (µg/mL), M (P_25_, P_75_)	9.65 (2.63, 22.77)	0.15 (0.10, 0.50)	< 0.001
Serum creatinine (µmol/L)	134.90 (104.15, 177.18)	67.20 (53.00, 139.00)	0.002
Blood lactate (mmol/L)	6.90 (4.23, 13.15)	2.30 (1.30, 3.40)	< 0.001
APACHE II score	10.00 ± 4.64	5.96 ± 3.95	0.003

SD: standard deviation; PCT: procalcitonin; APACHE II: acute physiology and chronic health evaluation II

The normality of continuous variables was examined by Shapiro-Wilk test. The continuous variables that confirmed to normal distribution were expressed as mean ± standard deviation (SD), and compared by independent sample t-test. The continuous variables with skewed distribution were expressed as median (P25, P75), and compared by the Mann-Whitney U test

**Fig. 1:**
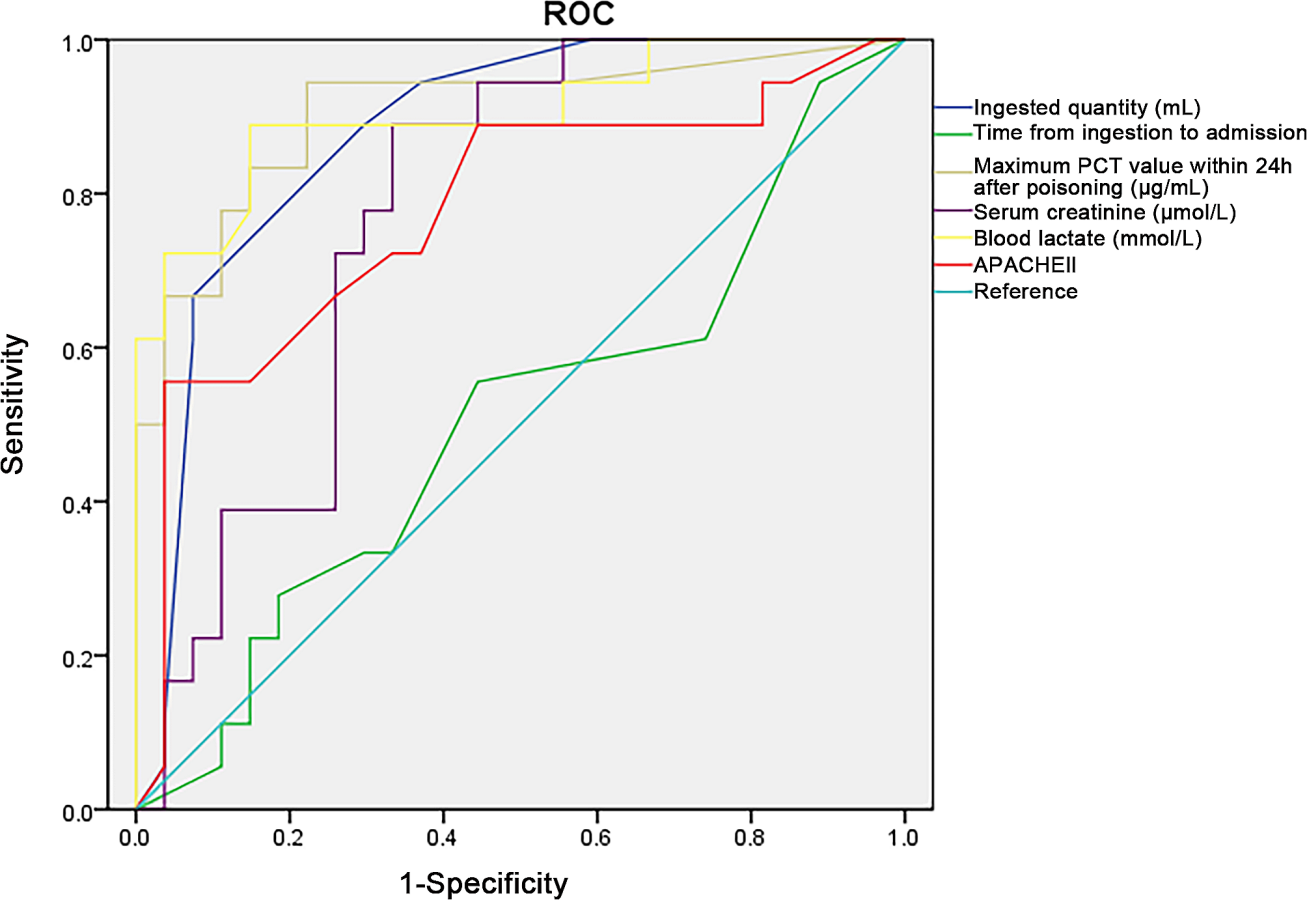
Predictive value for prognosis of diquat poisoning

The AUC of ROC indicated that the maximum PCT value within 24 h had surpassed predictive value (AUC = 0.905, 95% CI: 0.808-1.000, sensitivity: 94.4%, specificity: 77.78%) compared to the ingested quantity (AUC = 0.879, 95% CI: 0.776–0.981, sensitivity: 88.89%, specificity: 70.37%), serum creatinine (AUC = 0.776, 95% CI: 0.640–0.912, sensitivity: 88.90%, specificity: 66.70%), or APACHE II score (AUC = 0.778, 95% CI: 0.631–0.925, sensitivity: 55.60%, specificity: 96.30%). And the predictive value of the maximum PCT value within 24 h was comparable with blood lactate (AUC = 0.904, 95%CI: 0.807-1.000, sensitivity: 88.90%, specificity: 85.20%) **(**Fig. 1**and** Table 2**)**.

**Table 2:** Predictive value for prognosis

Characteristics	AUC	*P*	95% CI		Optimal cut-off value	Youden Index	Sensitivity (%)	Specificity (%)
			Lower Limit	Upper Limit				
Ingested quantity (mL)	0.879	< 0.001	0.776	0.981	80	0.593	88.89	70.37
Time from ingestion to admission (hours)	0.507	0.935	0.330	0.684	4.5	0.111	55.56	55.56
Maximum PCT value within 24 h after poisoning (µg/mL)	0.905	< 0.001	0.808	1.000	0.51	0.722	94.44	77.78
Serum creatinine (µmol/L)	0.776	0.002	0.640	0.912	85.65	0.556	88.90	66.70
Blood lactate (mmol/L)	0.904	0.000	0.807	1.000	3.75	0.741	88.90	85.20
APACHE II score	0.778	0.002	0.631	0.925	10.5	0.519	55.60	96.30

AUC: area under curve; CI: confidence interval; PCT: procalcitonin; APACHE II: acute physiology and chronic health evaluation II

## Discussion

The maximum PCT value within 24 h after poisoning might be a good predictor for the prognosis of patients with acute diquat poisoning. The findings concludes that monitoring PCT levels might help evaluate the prognosis of patients with acute diquat poisoning, especially when the dosage is uncertain.

The given results show that the PCT value within 24 h is a stronger predictor compared to other factors, such as the ingested quantity, serum creatinine, or APACHE II score, for diquat poisoning. The AUC of the ROC analysis was used to assess the discriminatory power of a diagnostic or predictive test. In this case, the AUC values for each factor indicate their predictive abilities. According to the results, the maximum PCT value within 24 h indicates a strong predictive value. The AUC for the ingested quantity for serum creatinine and APACHE II score suggest that while these factors have some predictive value, they are not as strong as the maximum PCT value. Interestingly, the maximum PCT value within 24 h had comparable predictive value to blood lactate, suggesting that both factors are equally good at predicting the diquat poisoning. These results are in accordance with previous reports. For instance, a study investigated the predictive value of various factors in sepsis and found that maximum PCT value had a strong correlation with disease severity and predicted patient outcomes with high accuracy [21]. Another study conducted a ROC analysis for comparing the predictive abilities of various factors for bacterial infection. They found that maximum PCT value had the highest AUC value, indicating its superior predictive ability [22].

ROC curve analysis demonstrated that arterial lactate had a good predictive power in evaluating the prognosis of patients, suggesting that in hospitals without facilities to test plasma paraquat concentration, measuring arterial lactate may be a simple and practical tool for assessing the severity of paraquat poisoning [23]. Another study found that APACHE II score, severity index of paraquat poisoning (SIPP), and serum lactate levels were effective predictors of prognosis where lactate levels showed the most accuracy in predicting mortality [24]. The findings of all these studies show that while other factors have the potential of predicting paraquat poisoning, PCT is a superior predictor, especially when the dosage is uncertain. After entering the body, diquat undergoes a redox reaction and forms a radical cation, which reacts with oxygen and generates reactive oxygen species (ROS) such as superoxide radicals. These ROS cause damage to cellular components, including lipids, proteins, and nucleic acid [25, 26]. Diquat poisoning causes an inflammatory response in the body, leading to the secretion and expression of serum inflammatory factors like tumor necrosis factor-alpha (TNF-α), interleukin-1beta (IL-1β), interleukin-2 (IL-2), interleukin-4 (IL-4), and interleukin-6 (IL-6) [27, 28]. According to Huang et al. [16], PCT, a secondary inflammatory mediator, increases as the degree of poisoning worsens. This results in more severe tissue cell damage and an earlier and more apparent systemic inflammatory response. In some cases, this can even lead to multiple organ dysfunction syndrome (MODS), with varying levels of PCT elevation. During bacterial infections, tissues other than the thyroid, such as the liver, lungs, and intestines, produce PCT. Since these tissue cells do not have secretory granules and conversion enzymes, PCT is released directly into the blood, causing significantly increased serum concentrations [29]. An observational study involving 4,858 patients found that largest increase in PCT was associated with *Escherichia coli* and other *Enterobacteriaceae bacteremia *[30]. In patients with diquat poisoning, particularly severe cases with a build-up of fluid in the intestine and paralytic ileu [31], bacteria from the intestine may enter the bloodstream, leading to infections and a significant increase in PCT levels. PCT is a relatively specific inflammatory marker for sepsis, helping in the early detection of bacterial infections and assessing disease severity [32-34]. High levels of PCT (especially > 10 µg/L) suggest a higher likelihood of Gram-negative bacterial infection[34]. If the PCT concentration exceeds 10 µg/L and continues to rise or does not decrease after treatment, it strongly indicates severe bacterial sepsis or septic shock, often accompanied by organ failure, which indicates a high risk of mortality [35, 36]. In a case report, a consumption of 160 mL diquat by a 30-years old male resulted in death due to multiple organ failure, particularly severe respiratory failure, with clear pulmonary lesions[16]. In another study, diquat poisoning led to increased levels of potassium, carbon dioxide, alanine aminotransferase, and aspartate aminotransferase in the mouse model. The study also included 29 human patients with diquat poisoning, who showed decreased levels of potassium and chloride, which led to death. The dead patients had higher levels of alanine aminotransferase, aspartate aminotransferase, blood urea nitrogen, and amylase, and high neutrophil-to-lymphocyte ratio [37]. These studies suggest that a combination of certain factors could accurately predict the outcomes of patient in diquat poisoning.

This study has several limitations. First, the study is limited by small sample size and limited indicators. These indicators lack correlation analysis between PCT and other indicators as well as prognosis. However, it is important to note that the PCR monitoring holds some positive value as an early prognostic indicator for patients with acute diquat poisoning patients. Second, this study was conducted in a single hospital, and thus limits the generalizability of the results to other settings or populations. Third, the retrospective design of the study and relying on medical records for data collection may lead to missing or incomplete data, as well as potential errors in data recording. Fourth, the study did not account for potential confounding factors that may influence the outcomes, such as concomitant medications and individual variations in metabolism.

## Conclusions

In conclusion, the maximum PCT value within 24 h after poisoning might be a good predictor for the prognosis of patients with acute diquat poisoning. Larger studies are needed to confirm these findings and explore potential prediction models involving PCT and other clinical parameters.

## Data Availability

All data generated or analysed during this study are included in this article.

## Abbreviations

PCT: Predictive value of procalcitonin
AUC: Area under the ROC curve
APACHE II: Acute physiology and chronic health evaluation II
CRRT: Continuous renal replacement therapy
SD: Standard deviation
ROC: Receiver operating characteristic
SIPP: Severity index of paraquat poisoning
ROS: Reactive oxygen species
TNF-α: Tumor necrosis factor-alpha
IL-1β: Interleukin-1beta
IL-2: Interleukin-2
IL-4: Interleukin-4
IL-6: Interleukin-6
MODS: Multiple organ dysfunction syndrome
